# Arylenevinylene Oligomer-Based Heterostructures on Flexible AZO Electrodes

**DOI:** 10.3390/ma14247688

**Published:** 2021-12-13

**Authors:** Anca Stanculescu, Marcela Socol, Oana Rasoga, Carmen Breazu, Nicoleta Preda, Florin Stanculescu, Gabriel Socol, Loredana Vacareanu, Mihaela Girtan, Aleksandr S. Doroshkevich

**Affiliations:** 1 Optical Processes in Nanostructured Materials Laboratory, National Institute of Materials Physics, 405A Atomistilor Street, P.O. Box MG-7, 077125 Magurele, Romania; marcela.socol@infim.ro (M.S.); oana.rasoga@infim.ro (O.R.); carmen.breazu@infim.ro (C.B.); nicol@infim.ro (N.P.); 2 Faculty of Physics, University of Bucharest, 405 Atomistilor Street, P.O. Box MG-11, 077125 Magurele, Romania; fstanculescu@fpce1.fizica.unibuc.ro; 3 Optical Processes in Nanostructured Materials Laboratory, National Institute for Laser, Plasma and Radiation Physics, 409 Atomistilor Street, P.O. Box MG-36, 077125 Magurele, Romania; gabriel.socol@inflpr.ro; 4 Electroactive Polymers and Plasmochemistry, P. Poni Institute of Macromolecular Chemistry, 41 A Gr. Ghica Voda Alley, 700487 Iasi, Romania; sloredana@icmpp.ro; 5 Laboratoire LPHIA, Université d’Angers, LUNAM 2, Bd. Lavoisier, 49045 Angers, France; mihaela.girtan@univ-angers.fr; 6 Frank Laboratory of Neutron Physics, Joint Institute for Nuclear Research, 6 Joliot-Curie Str., 141980 Dubna, Russia; doroh@jinr.ru

**Keywords:** organic semiconductors, arylenevinylene oligomers, flexible heterostructures, ZnPc, TPyP, AZO

## Abstract

We investigated the optical and electrical properties of flexible single and bi-layer organic heterostructures prepared by vacuum evaporation with a p-type layer of arylenevinylene oligomers, based on carbazole, 3,3′ bis(N hexylcarbazole)vinylbenzene = L13, or triphenylamine, 1,4 bis [4 (N,N’ diphenylamino)phenylvinyl] benzene = L78, and an n-type layer of 5,10,15,20-tetra(4-pyrydil)21H,23H-porphyne = TPyP. Transparent conductor films of Al-doped ZnO (AZO) with high transparency, >90% for wavelengths > 400 nm, and low resistivity, between 6.9 × 10^−4^ Ω·cm and 23 × 10^−4^ Ω·cm, were deposited by pulsed laser deposition on flexible substrates of polyethylene terephthalate (PET). The properties of the heterostructures based on oligomers and zinc phthalocyanine (ZnPc) were compared, emphasizing the effect of the surface morphology. The measurements revealed a good absorption in the visible range of the PET/AZO/arylenevinylene oligomer/TPyP heterostructures and a typical injection contact behavior with linear (ZnPc, L78) or non-linear (L13) J-V characteristics in the dark, at voltages < 0.4 V. The heterostructure PET/AZO/L78/TPyP/Al showed a current density of ~1 mA/cm^2^ at a voltage of 0.3 V. The correlation between the roughness exponent, evaluated from the height-height correlation function, grain shape, and electrical behavior was analyzed. Consequently, the oligomer based on triphenylamine could be a promising replacement of donor ZnPc in flexible electronic applications.

## 1. Introduction

Organic semiconductors offer a great potential for applications in plastic electronics and photovoltaics as an alternative to inorganic semiconductor-based devices, mostly because of their chemical flexibility, processability on large, flexible areas, and lower production cost [1,2,3,4]. Lately, there have been investigations of the optical and electrical properties of different types of organic heterostructures. The research interests included the bi-layer heterostructures based on two materials from small molecules, oligomeric or polymeric compounds, with different electron affinities (EA) (lower unoccupied molecular orbital (LUMO)) and ionization potentials (IP) (higher occupied molecular orbital (HOMO)).

Phthalocyanines are a group of compounds mostly used to prepare layers with holes conduction for a large area of applications. For example, a bilayer heterojunction based on copper phthalocyanine and fullerene C_60_ has been recently proposed as an active layer in organic photodetectors for red light [5]. Besides the advantages shown by the metal phthalocyanine donors, an important drawback is represented by the effect of the metal phthalocyanine (including ZnPc) oxide electrode interface states on the charge transport [6]. The aim of this paper is to propose new p-type conduction in organic compounds for replacing zinc phthalocyanine (ZnPc) in bi-layer ZnPc/5,10,15,20-tetra(4-pyrydil)21H,23H-porphyne (TPyP) flexible heterostructures deposited on zinc oxide doped with Al (AZO), for organic devices applications. AZO is a cheap, non-toxic, low electrical resistance transparent conductive electrode. Thin films from these compounds are easy to prepare by conventional methods, such as vacuum evaporation and deposition. It is also anticipated that their interface with an oxide electrode will not negatively affect the charge carrier transport, as is the case of the interface between the most used p-type conduction organic semiconductor ZnPc and oxide electrode.

In this context, are investigated two oligomers containing electron-donating groups based on aromatic amines, such as triphenylamine and carbazole [7,8,9]. Zinc phthalocyanine shows the tendency to form highly-ordered layers, which has a good influence on the properties of any device [10], will be the reference p-type conduction material for comparison with the mentioned oligomers. Previous studies have revealed the properties of heterostructures with a ZnPc layer deposited by the laser technique [11,12,13]. The use of ZnPc seems to be advantageous, but the central metal atom can have an important influence on the electronic properties of the organic ligand involved in the interaction between ZnPc and the surface of the oxide used as a transparent conductor electrode [6]. Some studies have shown an inhomogeneous charge transfer within the ZnPc layer deposited on an oxide layer [6]; this process affects the electrical properties of the organic heterostructures. Therefore, it is justified to analyze the possibility of using some metal-free organics such as arylenevinylene compounds as an alternative for replacing ZnPc in organic heterostructures. For example, the charge transfer process is favored by the fast electronic delocalization in triphenylamine [14]. On the other hand, the use of the oligomers of the arylenevinylene compound is justified by their easy processability. Thin films can be deposited on varied substrates using different methods, including the deposition by vacuum evaporation used in this paper and the deposition from solution, which is an important advantage compared to ZnPc.

As an acceptor, we use a non-metallic porphyrin 5,10,15,20-tetra(4-pyrydil)21H,23H-porphyne (TPyP) showing electron conduction. The pyridyl groups determine an increased EA, and the extended π-conjugated system leads to light absorption in a wide spectral range [15]. The effect of morphology on the optical and electrical properties of TPyP thin films deposited by vacuum evaporation have already been previously investigated [16].

The fabrication of electronic devices has increased the interest in new high-performance transparent conductor electrodes using materials that are not critical and can be deposited using conventional methods friendly for the environment. The transparent conductor electrode (TCE), as a part of organic devices, must show good transparency in the desired spectral region and low resistivity. Hitherto, the most widely used TCE is Indium Tin Oxide (ITO) due to its high optical transparency and metallic conductivity. However, it also has some disadvantages, such as the high refractive index and too low work function (WF). Therefore, it is difficult to assure an efficient injection of holes in organic semiconductors characterized by low-lying HOMO levels. On the other hand, indium is expensive, and its natural resources are limited [17,18,19,20,21]. The new perspective of cheap and non-polluting technologies for large-area flexible devices opens the search for other materials with adequate properties and compatible with flexible substrates to be used as TCE. Thus, in the attempt to replace ITO, many other metallic oxides have been investigated in single and multi-layer configurations (metallic oxide/metal/metallic oxide).

Lately, an increased interest has been shown for the use of zinc oxide (ZnO) as an electrode material, a II–VI semiconductor compound that is inexpensive and easy to prepare, characterized by a wide and direct bandgap (E_g_ ~3.2–3.4 eV). The properties of ZnO are affected by intrinsic defects (void/interstitial of Zn or O, or anti site O) forming either acceptor or donor levels in the bandgap. By extrinsic doping with Al, introduced interstitially or substitutionally for Zn, the space around the defect is changed, and the layers of Al-doped ZnO (AZO) are characterized by a good transparency and electrical conductivity [22,23,24]. Different methods have been tested for the deposition of ZnO doped with Al (or In), such as sol-gel [25,26] and co-sputtering of ceramic targets [27], emphasizing the effect of dopant chemical state [25] and dopant excess [27] on the doping efficiency. Other deposition methods include pulsed laser deposition [28], spin coating [29], reactive pulsed laser deposition [30], the effect of deposition conditions on the morphological, structural, optical, and electrical properties of the film being investigated.

However, the information related to metal oxide formation, characterization, and interfaces phenomena are important not only for organic electronics, sensors, and energy generation, but also for specific applications in energy storage (in the area of metal-ion batteries, supercapacitors). Thus, besides the use as transparent conductor electrodes, the metallic oxides have become interesting for other areas of application, such as electrode materials for supercapacitors. For example, binary metal oxides, such as cobalt oxide and molybdenum oxide, have been studied as materials for pseudocapacitor electrodes with improved properties in energy storage devices [31]. In the context of energy storage, lithium-sulfur batteries have attracted much research interest because of their advantages related to the theoretically anticipated high storage capacity, abundant resources, and environmentally friendly technology [32]. Numerous metal oxides have been tested with the purpose of surpassing the drawbacks associated with this type of battery, but the results are less encouraging compared to the use of TiN nanomaterials as cathode materials in energy storage devices [32].

The substrate is the base on which the device is built, and, in flexible electronics, many alternative substrates have been proposed to respond to the requirements arising from the manufacturing processes and operation conditions [33]. Paper is cheap, flexible, and biodegradable, but it shows high roughness, porosity, vapor permeability, and poor resistance to moisture. A more attractive alternative is represented by polymeric substrates, among which polyethylene terephthalate (PET) is the most used because of its high transparency, flexibility, solvent resistance, low price, and dimensional stability in high temperature [34]. Additionally, PET shows excellent water resistance, which is very important, especially for organic devices [34,35]. In this study, a commercially available polymer of the polyester family, PET, is selected as a flexible substrate for the deposition of heterostructures. PET is the substrate used for the successive deposition of the next layers and is an electric insulator. It can affect not only the morphology of the next deposited layer because of the particularities of its surface but, through the morphology, the properties of the deposited layers and heterostructures. Till now, PET has been intensively investigated for displays, organic light-emitting devices, and resistive touch-screens applications [36].

Among the above-mentioned properties of the substrate, porosity, dimensional stability, and thermal stability are very important. The porosity of the substrate could be important in printing technology using inks because the presence of a porous substrate can affect the ink spread, accuracy, and precise definition of printed circuit elements [37]. The performances of the devices are also determined by the surface energy and absorbance of the substrate [34] because these surface properties affect the ink spread and penetration, and ink layer thickness, finally affecting the resolution of the printed pattern [34]. PET dimensional stability in high temperature is also very important because this parameter reflects how the substrate reacts to environmental changes, a poor dimensional stability being correlated with the appearance of cracks and discontinuities in the printed pattern [34].

PET as a substrate for organic heterostructures is a polymer with a melting point temperature higher than 250 °C; beyond this temperature, it starts degradation [36,38]. The oligomers have been previously investigated from the point of view of their thermal stability [39]. The DSC measurements have revealed for both oligomers, endothermic peaks assigned to melting process at 212 °C (L13) and 170 °C (L78) and a stability for temperature <260 °C for L13 and <215 °C for L78 [39]. Thin films of TPyP and ZnPc have also been obtained by vacuum evaporation [16,40]. Thus, vacuum evaporation is an adequate method for the deposition of the selected organic layers and the realization of the proposed heterostructures.

Methods such as magnetron sputtering have already been tested for the deposition of a metal oxide layer on a polymeric substrate [41,42,43,44]. In the deposition processes involving continuous bombardment with energetic particles, the substrate temperature can be increased by tens of degrees but remains under the maximum service temperature [34] and glass temperature, T_g_ [45], for PET. This is the case of the deposition of Al-doped ZnO on PET by pulsed laser deposition, as mentioned below. This is important because, above the glass temperature, T_g_ [45], the combined effect of humidity and high temperature can lead to considerable reliability problems. However, below the T_g_, even in humid conditions, the reliability of PET was found to be excellent, even under prolonged exposure [46]. On the other hand, vacuum evaporation is not a process involving energetic particles, and therefore, PET covered by AZO is not affected during this deposition process.

Thus, selection of substrate, materials for component layers of the heterostructures, and deposition methods for the realization of the flexible heterostructures, are justified.

**Figure 1:**
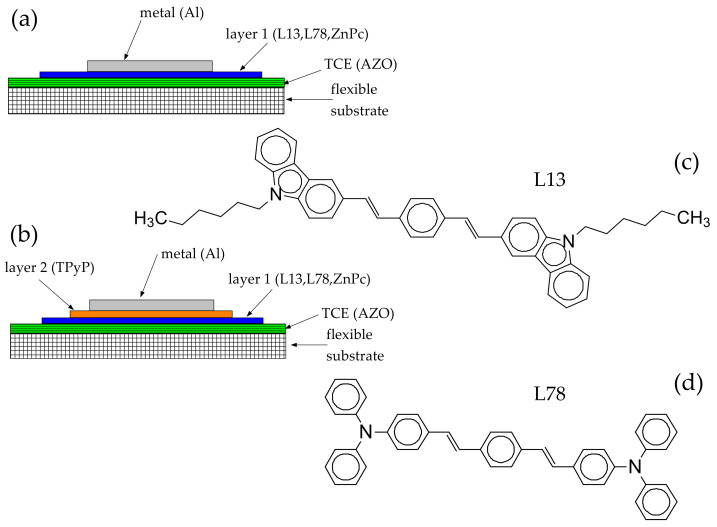
Organic heterostructures with single-layer organic (**a**), and bi-layer organic (**b**). Chemical structures of L13 (**c**) and L78 (**d**).

New single (Figure 1a) and bi-layer (Figure 1b) organic heterostructures have been prepared on flexible substrates of polyethylene terephthalate covered by AZO using arylenevinylene oligomers as p-type materials and TPyP as n-type materials and, the optical and electrical properties of these heterostructures have been discussed in correlation with the surface topography and energetic barriers at interfaces. The height-height correlation function (HHCF) was used for a statistical analysis of atomic force microscopy images and roughening study of the layers’ surface, emphasizing the correlation between the HHCF parameters and the properties of the layers. The main contributions of this paper refer to the use of donor thin films from arylenevinylene oligomers in heterostructures with potential electronic applications. These oligomers show good absorption in the visible spectral range and good transport properties of the charge carrier at the interface with the AZO electrode.

## 2. Materials and Methods

The arylenevinylene oligomers 3,3-bis(N-hexylcarbazole)vinylbenzene = L13 (Figure 1c) and 1,4-bis[4-(N,N-diphenylamino)phenylvinyl]benzene = L78 (Figure 1d) were synthesized by Wittig condensation starting from N-hexyl-3-formylcarbazole and 4-formyltriphenylamine, as described in previous papers [9,47].

The electrode of AZO was deposited by pulsed laser deposition (PLD) [48] on polyethylene terephthalate-PET substrates with an area of 24 mm × 24 mm and thickness of ~95 μm. The pulsed beam of a KrF * laser source, Coherent CompexPro 205 (Coerent LaserSystem GmbH & Co. KG, Gottingen, Germany) (λ = 248 nm, FWHM ~25 ns, repetition rate = 10 Hz) [49] was focused on the solid bulk target of ZnO doped with 2% Al produced by SCI Engineered Materials (Columbus, OH, USA). All samples were prepared using the same target in similar deposition conditions and geometrical configurations. The local deterioration of the target under the effect of the laser beam can cause variations in the flux of molecules, which could determine variations in the properties of the AZO films. Therefore, the target was rotated to reduce the local deterioration, which could affect the quality of the deposited layer. The material was ablated at a fluence of 2 J/cm^2^, using 20,000 pulses, in an atmosphere of oxygen at a pressure of 1 × 10^−2^ mbar and was subsequently deposited onto the substrate kept at room temperature and situated at a distance of 8.5 cm from the target.

The vacuum evaporation method was previously used for the deposition of organic thin films of ZnPc and TPyP [16,39,50]. In this study, we used this method to prepare organic heterostructures (Table 1) with a single layer of ZnPc, L13, and L78 (Figure 1a) and a bi-layer based on L13, L78, and ZnPc as the donor and TPyP as the acceptor (Figure 1b). The pressure in the deposition chamber varied between 1.4 × 10^−5^ mbar and 0.9 × 10^−5^ mbar, and the evaporation temperature was approximately 195 °C for L78, 170 °C for L13, 210 °C for ZnPc, and 180 °C for TPyP. The thickness of the organic films varied between 190 nm and 240 nm and was measured during the deposition process using a monitoring system based on quartz crystal, SQC-300 Deposition Controller produced by Sigma Instruments (Cranberry Township, PA, USA).

The top metallic electrode of aluminum (Al) was deposited by vacuum evaporation using SPECTROS equipment (Kurt J. Lesker St. Leonards-on-Sea, East Sussex, UK) and specially designed circular masks. The pressure in the deposition chamber varied between 1 × 10^−6^ mbar and 5 × 10^−6^ mbar, and the deposition rate was 4 Å/s. The Al contact had an area of 0.28 cm^2^ and a final thickness, measured with a thickness monitor with quartz crystal, of 150 nm.

Information about the morphology of AZO layers was obtained by scanning electron microscopy (SEM) performed with an EVO 50 XVP microscope (Carl Zeiss Microscopy Deutschland GmbH, Oberkochen, Germany): V_acc_ = 20 kV, working distance = 20 mm; magnification = 145 kX. The scanning electron microscope Zeiss EVO 50XVP equipped with the Energy Dispersive X-Ray (EDX) analyzer QUANTAX (Bruker, Billerica, MA, USA) working at 20.00 kV and magnification of 0.78 kX, was used to analyze the elemental composition of the AZO films.

For all samples, the signal was collected in the same measurement conditions from a well-delimited area (407 × 259 μm^2^) imaged by SEM, and the obtained value is an average concentration over this area. EDX analysis revealed a composition of 1.8 (at. %) Al in AZO deposited on PET (Table 2).

Atomic force microscopy (AFM) with a 4000 MultiView System (Nanonics Imaging Ltd., Jerusalem, Israel) was used to obtain details on the roughness of the surface of the flexible substrate, AZO, and organic films. The surfaces were scanned in tapped mode working in phase feedback, with Cr/Al coated-glass tuning fork-type probes having a diameter of 20 nm, using the following parameters: scanning area = 10 μm × 10 μm, scan resolution = 256 lines, scanning speed = 6.12 lines/s, resonance frequency = 38 kHz, and quality factor = 1715 for organic films and, resonance frequency = 35 kHz and quality factor = 1530 for AZO films.

The structural particularities of the AZO layers were investigated by X-ray diffraction with a D8 Advance Diffractometer (Bruker, Billerica, MA, USA) using the Cu K_α_ line, working in locked-coupled mode (V_acc_ = 40 kV, I_anode_ = 40 mA, increment = 0.04° and scan speed = 1 s/step).

The UV-VIS transmission spectra of flexible substrates and AZO films were recorded with a double beam 10e CINTRA Spectrophotometer (GBC Scientific Equipment, Braeside, VIC Australia) and those of the organic films with a UV-Vis-NIR 5000 Carry Spectrophotometer (Agilent, Santa Clara, CA, USA). A F-900 Spectrofluorometer (Edinburgh Instruments Ltd., Livingston, UK) was used to draw the photoluminescence (PL) spectra: λ_ex_ = 335 nm, Δλ = 350–650 nm and λ_ex_ = 435 nm; Δλ = 450–800 nm.

The resistance of AZO films was measured in the four-point probe configuration [51], and the I-V plots of organic heterostructures were recorded in a transversal configuration with 3-wires contact geometry (1 contact on Al and 2 contacts on AZO) to remove the effect of the contacts [52], using a Keithley 2400 Source meter (Tektronix- Beaverton, OR, USA).

## 3. Results and Discussions

**Figure 2:**
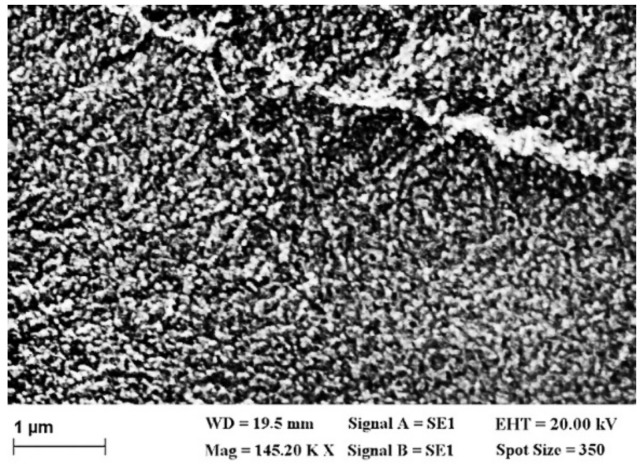
Typical SEM image of AZO layer deposited on PET substrate.

SEM images of AZO films deposited on PET substrates (Figure 2) have shown a morphology characterized by grains with irregular shapes and different dimensions, clusters of grains randomly dispersed, and defects, nonuniformities, associated with the disorder. The defects are generated by the differences between the thermal properties of the plastic substrate and AZO film, determining residual internal stress or by the stress developed in the AZO layer during the deposition process. The substrate surface defects such as scratches are preserved by the AZO layer. These defects can be centers for radiation or charge carriers scattering or/and recombination.

**Figure 3:**
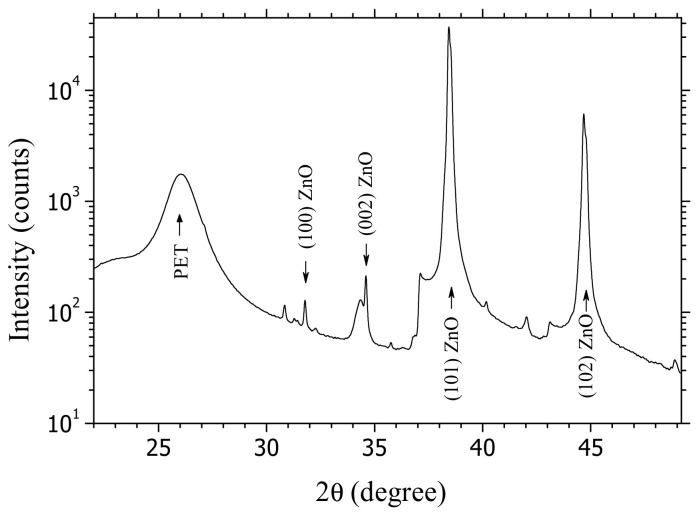
Typical XRD spectra for AZO layers deposited on PET substrate.

The XRD patterns (Figure 3) of AZO films deposited by PLD on a flexible substrate at room temperature revealed a broad diffraction peak centered at ~26°, which is associated with PET [53] flexible substrates. The film of AZO deposited on PET is polycrystalline, with the grains oriented in some well-defined directions. The peak situated around 2θ = 34.5° corresponds to reflections on the plane (002) according to the standard ZnO hexagonal structure [28,54] and confirms that a number of grains are c-axis oriented, perpendicular to the substrate. The strong peaks localized at 38.5° and ~45° correspond to reflections on the (101) plane [55] and (102) plane [56], respectively, and the weak peak localized at ~32° to reflections on the (100) plane [57]. The diffraction peaks can be slightly shifted compared to the references or widened because of the defects.

**Figure 4:**
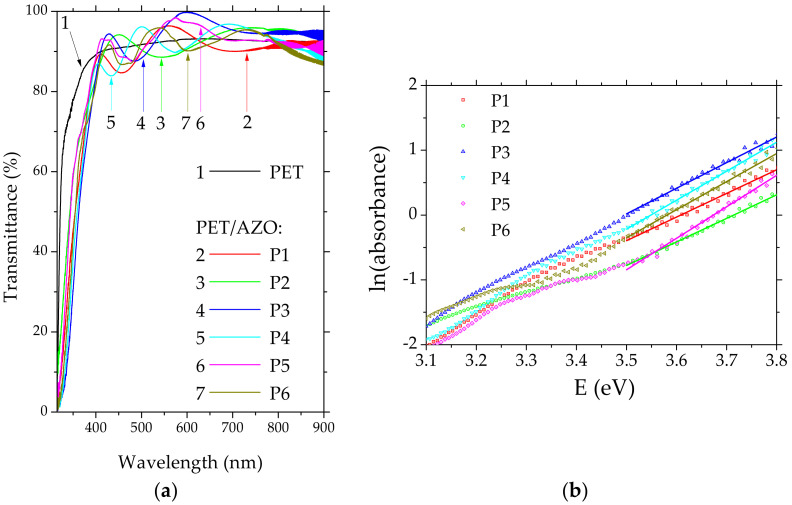

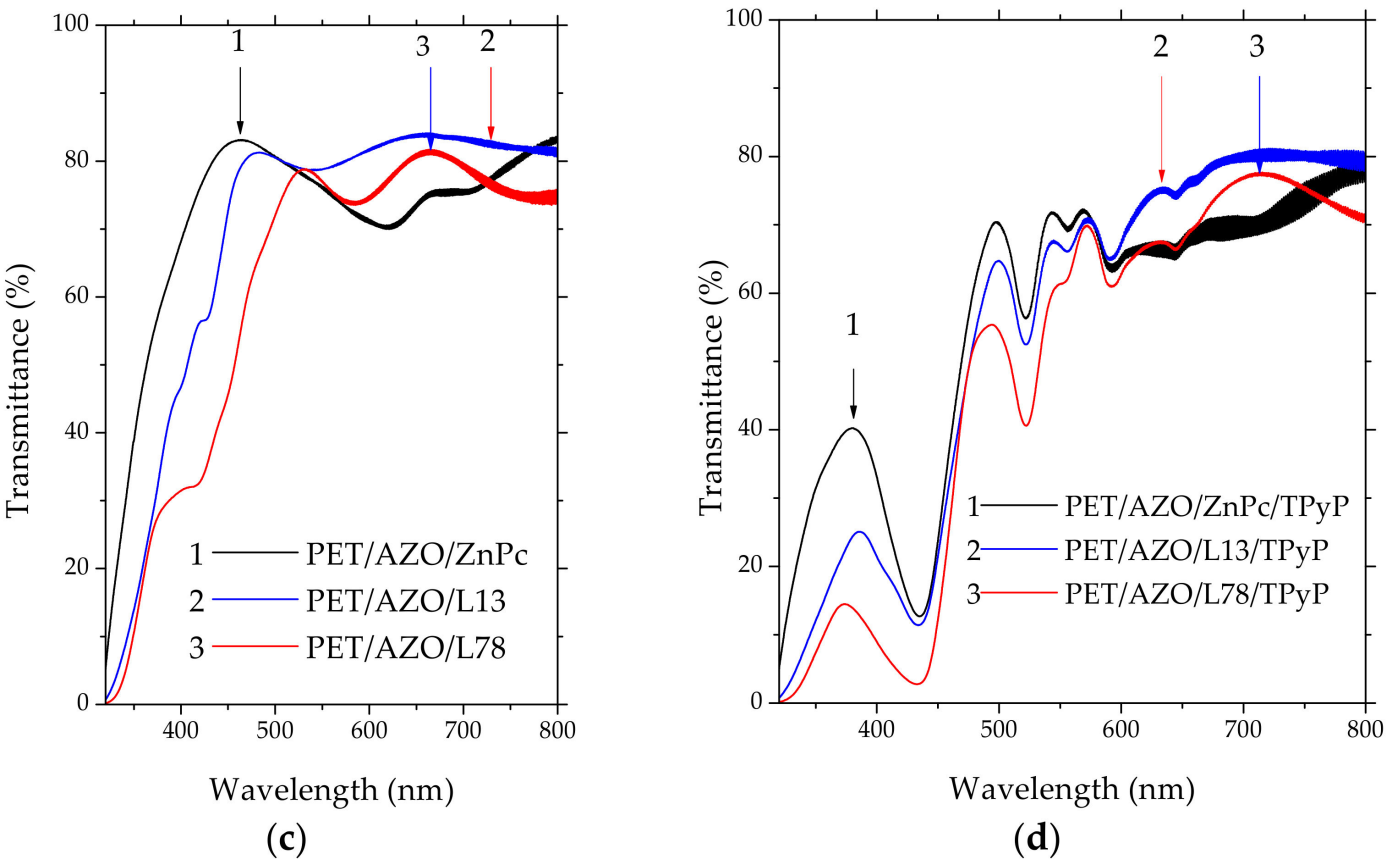
UV-Vis transmission spectra of AZO layer deposited on PET (**a**); single-layer organic heterostructures on PET/AZO (**c**); bi-layer organic heterostructures on PET/AZO (**d**). Urbach law for AZO layer deposited on PET (**b**). The substrates for (**c**,**d**) are indicated in Table 1.

The average transmission of PET substrate is high (~90%) at wavelengths over 400 nm (Figure 4a), because the regular reflectance (R_reg_) [58] at the interface PET/air is low, R_reg_ = 0.049, considering the refractive index of PET, n_PET_ = 1.57 [59]. The interference fringes situated in the NIR region are correlated with the interference phenomenon which appears inside the substrate. All the AZO films deposited by PLD on PET substrates were characterized by high average transparencies (~90%) in the spectral range 400–800 nm. The UV-Vis transmission curves of PET/AZO samples (Figure 4a) were drawn using the PET substrates as reference samples. The transmission of the structure PET/AZO is higher than the transmission of the PET substrate alone because AZO (n_AZO_ = 1.95 [60]) film acts as an anti-reflection layer. The R_reg_ at the interface PET/AZO (R_reg_ = 0.012) is much lower than R_reg_ at the interface PET/air (R_reg_ = 0.049).

For the evaluation of the thickness of the AZO layer deposited on PET (Table 1), we used the relationship between two successive extremes showed by the transmission spectra in the NIR region [61].

The experimental transmission data of AZO films also offer information about the degree of disorder. An exponential dependence between the absorption coefficient, α, and the photon energy, hν, for photon energy between 3 and 4 eV has been evidenced for AZO films deposited on PET (the Urbach law [62]):

(1) $${\alpha =\alpha }_{o}\times \exp\left(h\nu /E_{o}\right)$$

where α_o_ = constant and E_o_ = Urbach energy, which is correlated with the disorder. Large E_o_ values mean high compositional, morphological, and structural disorder in AZO films. The Urbach energy indicates the lack of long-range order in the layer and the presence of extended energetic levels in the forbidden gap below the absorption edge. By the extrapolation of the linear part of the curves ln(αd) versus (hν), where d = film thickness (Figure 4b), we obtained the E_o_ values, represented by the intercept with the OX axis. For AZO films deposited on PET, the E_o_ values were between 0.206 eV and 0.275 eV, with an average value of 0.244 eV corresponding to a significant degree of disorder. Thus, the PET/AZO influences the properties of the organic heterostructures because the disorder of the AZO layer deposited on plastic substrate induces disorder in the subsequently deposited layers affecting their morphology and finally their properties.

The UV-VIS transmission spectra of the organic layer of L78 and L13 deposited on PET/AZO revealed a weak absorption band centered around 420 nm, which is missing in the spectrum of the ZnPc layer deposited on PET/AZO (Figure 4c). This behavior is favored by the conformational flexibility of the compounds containing carbazole (L13) or phenylamine (L78) aromatic units connected by vinyl segments. This molecular structure assures the delocalization and superposition of the molecular orbitals and determines the electronic intra-molecular interactions and strong polarization interactions with the surrounding molecules [63]. The peaks situated around 640 nm and 720 nm in the UV-Vis spectra of the heterostructures realized with ZnPc arise from the π-π* transitions in the phthalocyanine macrocycle. The shape of the transmission spectra of the heterostructures with bi-layer organic (Figure 4d) is determined mostly by the behavior of the TPyP, which is a porphyrin characterized by the presence of some well-defined absorption bands in the visible range. All the bi-layer heterostructures have revealed the Soret band (B) situated at 450 nm and four (Q) bands situated at 520 nm, 555 nm, 590 nm, and 640 nm. The slight red shift of the absorption peaks in the TPyP films compared to the reference peaks for TPyP in solution is a consequence of the stronger interactions between the molecules in the solid state. The strong light absorption favors the process of exciton generation as a preliminary condition for the appearance of the free charge carriers.

**Figure 5:**
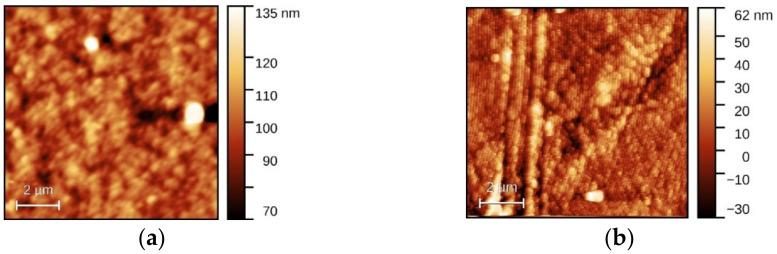
Typical AFM images for PET (**a**) and PET/AZO (**b**).

**Figure 6:**
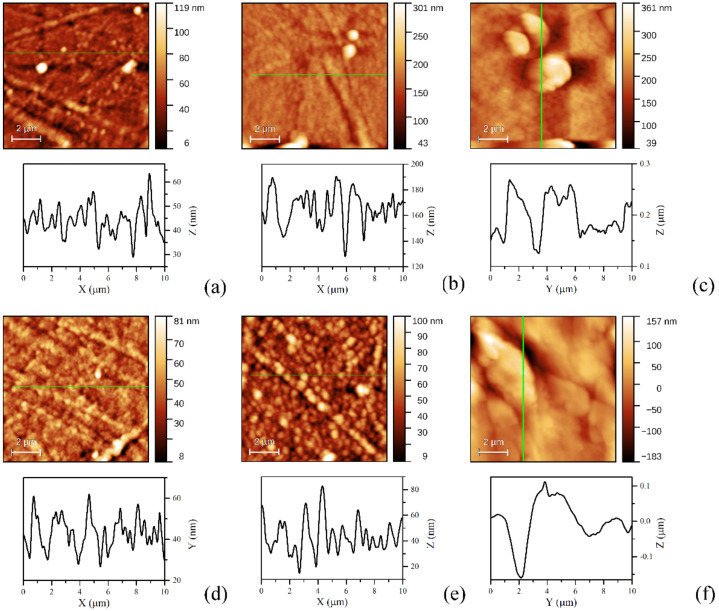
Typical AFM images and profile line for PET/AZO/ZnPc (**a**), PET/AZO/L13 (**b**), PET/AZO/L78 (**c**), PET/AZO/ZnPc/TPyP (**d**), PET/AZO/L13/TPyP (**e**), PET/AZO/L78/TPyP (**f**). The substrates are indicated in Table 1.

In addition to light absorption in films and reflectance at interfaces, the scattering on defects, including the scattering on the boundaries of grains/clusters, affects the properties of the organic heterostructures. In the first stage, the surface morphology of the flexible substrate, PET, is important because it influences the morphology of the AZO film covering the substrate. The values of the surface amplitude parameters, namely roughness mean square (RMS) and roughness average (RA), are presented in Table 3. The AFM images (Figure 5) have pointed out a roughness of RMS = 11.2 nm, RA = 7.3 nm for PET substrate (Figure 5a) and of RMS = 13.5 nm, RA = 9.6 for AZO layer on PET (Figure 5b). The AFM image of the AZO film deposited on PET has revealed a morphology characterized by grains and rarely and randomly distributed grains clustered with some weak ordering trend. The AFM images of the single-layer organic (Figure 6a–c) have revealed an increase in roughness for the L13 film deposited on PET/AZO, RMS = 22 nm, RA = 13.6 nm (Figure 6b). The highest roughness was obtained for the L78 film deposited on PET/AZO, RMS = 43 nm, RA = 31 nm (Figure 6c). The lowest roughness was revealed by the ZnPc film deposited on PET/ AZO, RMS = 12.3 nm, RA = 8.3 nm (Figure 6a), and is comparable with the roughness of AZO deposited on PET (Figure 5b). This behavior can be explained by the more orderly arrangement of the planar molecules of ZnPc, which fit better in the “valleys” of the AZO surface compared to the large, non-planar molecules of L13 and L78, showing conformational flexibility. The films of ZnPc and L13 deposited on PET/AZO also showed a granular morphology characterized by grains and small dimension clusters of grains, while the film of L78 deposited on PET/AZO showed randomly distributed large dimension bubble-like clusters.

In the bi-layer organic deposited on PET/AZO (Figure 6d–f), the roughness of the TPyP supplementary layer deposited on top of the ZnPc and L13 was lower than the roughness of ZnPc and L13. TPyP deposited on ZnPc covering the PET/AZO substrate was characterized by RMS = 8 nm and RA = 6.2 nm (Figure 6d), and the TPyP deposited on the L13 covering the PET/AZO substrate was characterized by RMS = 13 nm, RA = 10.2 nm (Figure 6e). This behavior is determined mostly by the mechanism of accommodation of the molecules from the first organic layer (ZnPc or L13) on AZO deposited on PET substrates. Both the film of the TPyP deposited on the ZnPc and L13 present granular morphology with more clustering when TPyP is deposited on L13 and stronger orientation trend when TPyP is deposited on ZnPc. The highest roughness has been shown by the layer of TPyP deposited on L78 covering PET/AZO, RMS = 61 nm, RA = 46 nm (Figure 6f). This film showed a morphology with well-packed bubble-like clusters. The roughness was determined by the particularities of the growth process of the first layer of L78 on AZO. Thus, the highest roughness was shown by the single- and bi-layer samples realized with oligomer L78. Additionally, the molecule of TPyP fits well with the molecule of ZnPc, because of geometric similarities, determining the lowest roughness. When the molecules of TPyP are deposited over the film of planar ZnPc molecules, an order is imposed by the mesosubstituents, often named legs. These legs are able to rotate around the C-C σ bond and connect them to the core of the porphyrin [64]. Thus, it allowed the conformational adaptation of the TPyP molecules to their local environment. In turn, ZnPc molecule macrocycles preferentially lie parallel to the surface with their stacking axes inclining to the substrate [65,66]. In the case of a twisted molecule, such as the molecules of L13 and L78, the arrangement of the molecules does not respect any order when the molecules cover the ZnPc film.

The surface roughness determines different scattering mechanisms affecting the optical and electrical properties [67]. Therefore, the surface morphology, which is correlated with the layered growth process, is very important for specific device applications. The surface morphology can be studied with the amplitude parameters, such as RMS and RA, which have been evaluated from the AFM images using the Gwyddion software. However, the evaluated RMS and RA refer only to the vertical properties of the surface. Therefore, a more rigorous analysis of the film morphology is obtained by statistical analysis of the digitized information obtained from AFM images using the height-height correlation function (HHCF). This function reflects the correlation between the surface heights separately laterally and can be useful in identifying the type of growth between self-affine (characterized by smooth flat feature for large lateral distances) and mound-like (characterized by hillocks for large lateral distances) growth, as a result of the two competing processes, surface smoothing, and surface roughening.

The mean square surface fluctuation for a self-affine surface, $H\left(r\right)=\langle {\left[h\left(x\right)-h\left(x+r\right)\right]}^{2}\rangle$, where *h*(*x*) is the surface height at the position *x* on the scanned area, has the following expression [68,69]:

(2) $$H\left(r\right)=2w^{2}\left[1-\exp{\left(\frac{r}{\xi }\right)}^{2\alpha }\right]$$

where w represents the surface width and corresponds to the long-range roughness and is associated to RMS, α represents the roughness exponent and is correlated with the surface irregularities, and ξ represents the lateral correlation length, with the heights beyond this distance not being sign correlated. These parameters have been evaluated by fitting the function (2) with the HHCF calculated by Gwyddion software from the AFM profile of the prepared samples. The HHCF evolution reflects the behavior of the surface morphology during the growth process. In the first step, HHCF increases with lateral distance until it reaches a plateau. This region corresponds to an increased correlation between the surface heights separately laterally. The distance when the plateau begins is the coherence length ξ. In the second step, HHCF shows a plateau for distances larger than the coherence length. This plateau corresponds to the region where no correlation exists between the heights separated laterally, and the surface height variation is random. An oscillatory plateau region in the HHCF representation for lateral distances larger than ξ is associated with a mound-like surface morphology where the oscillation period is related to the separation between the mounds. In this region, the growth is dominated by a surface roughening process. A flat plateau region in the HHCF representation for lateral distances larger than ξ is associated with a smooth surface morphology. In this region, the growth is dominated by the smothering process of the surface [68,69].

**Figure 7:**
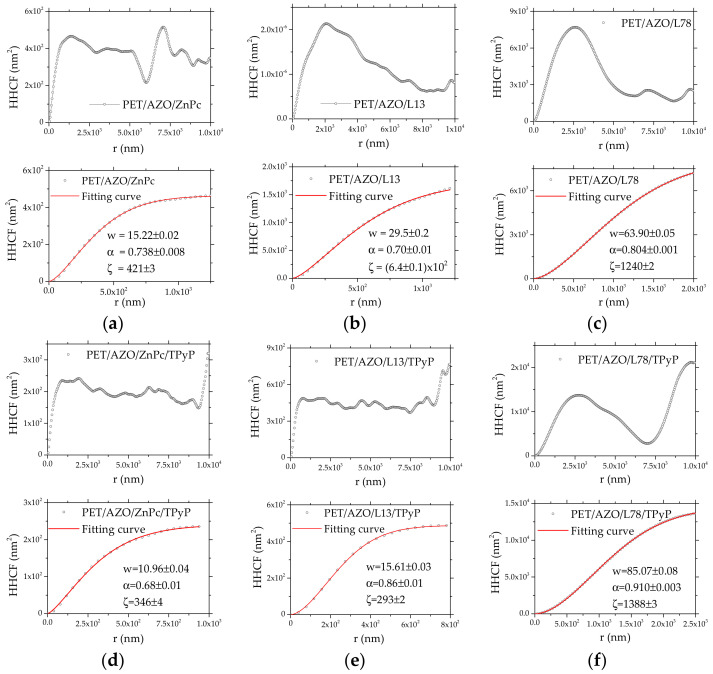
Height-height correlation function (HHCF) as a function of lateral distance, r, as obtained from the AFM surface profile scans, for large and small r: PET/AZO/ZnPc (**a**), PET/AZO/L13 (**b**), PET/AZO/L78 (**c**), PET/AZO/ZnPc/TPyP (**d**), PET/AZO/L13/TPyP (**e**), PET/AZO/L78/TPyP (**f**).

For our samples, the HHCF evolution reflects the behavior of the surface morphology during the organic film deposition by vacuum evaporation on AZO and on another organic film. Some single- and bi-layer organics such as ZnPc, ZnPc/TPyP, and L13/TPyP deposited on PET/AZO showed an oscillatory plateau region in the HHCF representation for large lateral distances, which is correlated with a mound-like surface morphology [68,69]. In the case of the ZnPc (Figure 7a) and TPyP layers deposited on ZnPc (Figure 7d), the w parameters were lower than the corresponding values of RMS, but are comparable, which means that the use of HHCF is adequate and the surface is affine. By adding the supplementary layer of TPyP, the RMS value decreased, which means that the layer was smoother than the ZnPc layer. However, the layer of TPyP deposited on ZnPc showed a lower roughness exponent, α, which corresponds to a rougher surface. This seems to be a contradiction [70].

A contradiction was also revealed when the TPyP layer was deposited on the triphenylamine-based oligomer, L78 (Figure 7f). In this case, the value of roughness exponent, α, very close to 1 indicates a very smooth surface, while the high value of RMS is correlated with a rougher surface. These apparent contradictions can be explained by the fact that the roughness exponent, α, is not a measure of roughness but is a quantification of how the roughness changes with the length scale [71]. On the contrary, no contradiction was revealed when the TPyP layer was deposited on the carbazole-based oligomer, L13 (Figure 7e). The roughness exponent increased, being correlated with a smoother surface in concordance with the decrease in RMS, which was also correlated with a smoother surface. The validity of HHCF analysis and the affine character of the surface was also confirmed in the case of TPyP deposited on L13 films, where the w parameter was slightly higher than the corresponding values of RMS but are comparable. In the case of L13 (Figure 7b), L78 (Figure 7c), and TPyP on the L78 (Figure 7f) deposited on the PET/AZO substrate, the difference between w and RMS was high, confirming that the surface of these layers was not affine.

The HHCF increased linearly with distance and reached a value approximately constant at distances larger than the coherence length, ξ, which was 1125 nm for the ZnPc film deposited on AZO, 810 nm for TPyP deposited on ZnPc, 1000 nm for L13 deposited on AZO, 600 nm for TPyP deposited on L13. The samples with L78 film deposited on AZO and TPyP film deposited on L78 showed the highest values for RMS and w and presented the highest values of the lateral correlation length, 1240 nm and 1388 nm, respectively. Thus, the supplementary film of TPyP reduced ξ, the distance within which any two heights on the surface are correlated for ZnPc and L13 films and increased ξ for L78.

On each sample, we drew the profile line and identified the shape of grains on the surfaces of the films because the type of grains affects the properties of the films. For example, the shape of the grain is correlated with the roughness exponent, α, which influences the charge transport properties in the film [72].

Thus, the film of ZnPc and L13 on PET/AZO showed a grain morphology (Figure 6a,b). By adding TPyP, it changed to slightly smaller and sharper grains morphology (Figure 6d,e). The profile line of the L78 film has revealed a large round grain morphology (Figure 6c). The morphology has been modified, by adding the layer of TPyP, in a morphology showing larger, nearly flat grains (Figure 6f).

**Figure 8:**
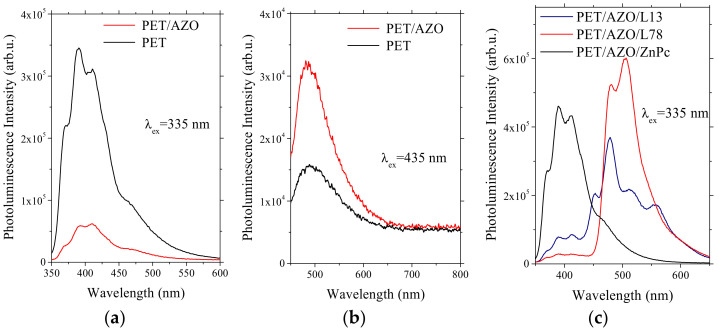

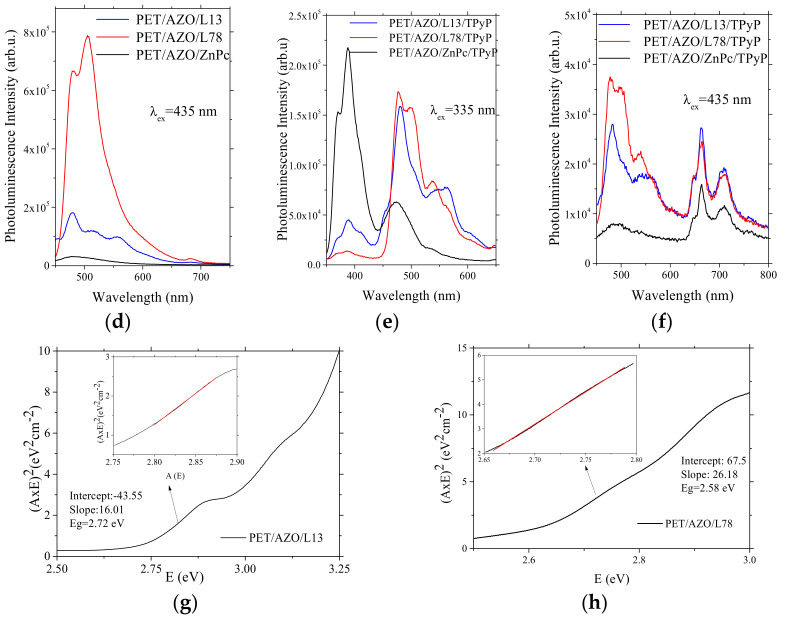
PL spectra of AZO layer deposited on PET substrate, λ_excitare_ = 335 nm (**a**). AZO layer deposited on PET substrate, λ_excitare_ = 435 nm (**b**). Single-layer organic on PET/AZO, λ_excitare_ = 335 nm (**c**). Single-layer organic on PET/AZO, λ_excitare_ = 435 nm (**d**). Bi-layer organic on PET/AZO, λ_excitare_ = 335 nm (**e**). Bi-layer organic on PET/AZO, λ_excitare_ = 435 nm (**f**). Tauc plot for L13 deposited on PET/AZO (**g**). L78 film deposited on PET/AZO (**h**), where A is absorbance and E is the photon energy. The substrates for (**c**–**f**) samples are indicated in Table 1.

At excitation with UV radiation (λ_ex_ = 335 nm), the flexible substrate has shown a broad structured emission band between 350 nm and 550 nm with three local maximums situated at 370 nm, 390 nm, and 420 nm, the last maximum showing a weak shoulder at 480 nm (Figure 8a). The shape of the emission spectra of the uncovered flexible substrate was preserved by the deposition of the AZO layer. The reduced intensity of the PL peaks of AZO covered compared to the uncovered flexible substrate (Figure 8a) can be correlated to the losses attributed to defect-related recombination. The emission was situated in the region characterized by significant disorder according to Urbach law (for λ > 310 nm). Another mechanism for radiation loss could be the re-absorption of the emitted radiation in the thickness of the AZO layer.

At excitation with λ_ex_ = 435 nm, the spectrum of flexible substrate showed a non-structured emission band centered on 490 nm. The substrates covered by the AZO also showed only a sharp band between 450 nm and 550 nm, with a maximum at ~490 nm (Figure 8b). The more intense peak in the AZO-covered substrate suggests overlap between the emission of the substrate and the emission associated with a recombination mechanism involving the Al dopant between the intrinsic donor and acceptor defects in AZO [73,74,75]. This peak situated at 490 nm, corresponding to a photon energy of 2.53 eV, can be assigned to a transition from the energetic level of Zn interstitial to Zn vacancies because this value is very close to the value of ~2.54 eV, evaluated theoretically for this transition [76].

At excitation with λ_ex_ = 335 nm, we have no evidence of supplementary PL peaks situated in the visible range for the structure with a single organic layer of ZnPc deposited on PET/AZO (Figure 8c). This means that the shape of the PL emission band is determined by the emission behavior of the substrate. The peak associated with the fluorescence by de-excitation from the higher singlet state (S_1_) to different vibrational levels of the ground excitonic state in ZnPc was also situated around 400 nm [77].

The heterostructures based on oligomers (L13 and L78) show, at excitation with λ = 335 nm, a broadened structured band between 400 and 650 nm with local maximums (Figure 8c). The molecular structure of L13, where carbazole units are introduced at the terminals of the conjugated backbone resulting in a less planar molecule, can determine a non-radiative decay and a decrease in PL intensity [78]. The spectrum of L13 film (Figure 8c) revealed a strong peak situated at 480 nm (E = 2.58 eV) and three weak peaks situated at 454 nm (E = 2.73 eV), 515 nm (2.41 eV), and 555 nm (E = 2.23 eV). After excitation with photons with an energy of 3.7 eV (335 nm), the molecules de-excited by non-radiative processes such as vibrational relaxation and internal conversion, IC (a crossover of two states with the same multiplicity) to the S_1_ level with an energy of 2.73 eV. This value corresponds to the first edge of the fundamental absorption (E = 2.72 eV) in L13, obtained applying the Tauc plot (Figure 8g). After that, the radiative de-excitation by fluorescence takes place, from the first excited singlet state (S_1_) to the fundamental state (S_0_), corresponding to the peak situated at 454 nm and blue fluorescence. The other emission peaks situated at 2.58 eV, 2.41 eV, and 2.23 eV were correlated with the radiative de-excitation from sub-band excitonic levels. The last peak was associated with the deep-level exciton de-excitation.

Considering the molecular structure of L78, due to the dihedral angle between the plane of the phenyl ring and the plane of the N-bonded C atoms in the triphenylamine-based oligomer, the molecule has the tendency to adopt a twisted configuration. This configuration can be correlated with a non-radiative decay determined by the geometrical relaxation [79] and a decrease in the PL intensity. The spectrum of L78 film (Figure 8c) revealed a strong peak situated at 506 nm (E = 2.47 eV) and a weaker one situated at 482 nm (E = 2.57 eV). After excitation with photons with an energy of 3.7 eV (335 nm), the molecules were de-excited by non-radiative processes like vibrational relaxation and IC from the energy level of 3.7 eV to the energy level of 2.57 eV. The subsequent radiative de-excitation by fluorescence from S_1_ to S_0_ corresponds to the peak situated at 2.57 eV and cyan-green fluorescence. This is in concordance with the edge of the fundamental absorption (E = 2.58 eV) in L78, obtained from the Tauc plot (Figure 8h). The other peak situated at 2.47 eV can be correlated with the radiative sub-band excitonic de-excitation.

The emission peaks of the L13 and L78 films showed structures corresponding to vibronic relaxation due to the coupling between the excitation transitions and the stretching vibrations [80]. The position of the emission band is the result of the delocalization over the entire conjugated backbone of the π-electron conjugation through the lone electron pair of the nitrogen atom of the carbazole or triphenylamine end-groups [78,79].

At excitation with λ = 335 nm, the structures with the bi-layer organic based on L78/TPyP showed PL bands centered at 485 nm, 510 nm, and 535 nm, those based on L13/TPyP showed PL bands centered at 485 nm and 570 nm with a weak shoulder at 545 nm, and those based on ZnPc/TPyP showed a PL band centered at 475 nm with a weak shoulder at 540 nm (Figure 8e). The presence of the second organic film of TPyP has determined the appearance of supplementary emission peaks at 475 nm in ZnPc, 387 nm and 575 nm in L13, and 535 nm in L78, peaks unsolved in the absence of TPyP (Figure 8c). The peaks situated under 400 nm are associated with the emission of AZO-covered flexible substrates.

At excitation with 435 nm, the PL spectrum of PET and PET/AZO (Figure 8b) revealed a large emission band centered on ~500 nm. The emission peaks situated at λ < 600 nm for both PET/AZO/L13 (Figure 8d) and PET/AZO/L78 (Figure 8d) were superimposed on the peak of PET/AZO. A strong peak situated at 480 nm and two weaker peaks situated at 513 nm and 555 nm were also revealed by the PL spectrum of L13 deposited on PET/AZO. A strong peak situated at 505 nm with the shoulder at 482 nm and a very weak emission band situated between 675 nm and 700 nm have been remarked on the PL spectrum of L78 deposited on PET/AZO. The PL of the ZnPc film (Figure 8d) showed a weak broadband with a maximum around 485–490 nm, the energy of 2.5 eV corresponding to radiative de-excitation from the triplet state T_1_ to the fundamental level, S_0_. By the absorption of the radiation of 435 nm (E = 2.85 eV), the molecule is excited on a higher vibrational level of the triplet state. From this level, the molecule non-radiatively de-excited by vibrational relaxation on the lowest vibrational level of T_1_ and then de-excited by radiative phosphorescence from T_1_ to S_0_.

At excitation with visible light λ = 435 nm, the emission spectra of the bi-layer organic heterostructures show, between 450 nm and 600 nm, a peak shape similar to that of the emission spectra at UV excitation and a supplementary broad emission band between 625 nm and 750 nm. This band shows a sharp peak situated at 665 nm, a broader one at 650 nm, and a lower peak situated at 715 nm (Figure 8f). The peaks situated at λ > 600 nm are attributed to TPyP because the free base TPyP revealed, at excitation with λ = 435 nm, two emission Q bands situated at 660 nm and 710 nm [15]. These peaks were superimposed on the absorption bands of TPyP, and thus, the emitted radiation could be partially reabsorbed on deep energetic levels. In comparison with the heterostructures with single- and bi-layer organics based on oligomers, the heterostructures based on ZnPc showed the lowest PL at excitation with visible wavelength.

Because many of these peaks are situated in the absorption regions of the heterostructures realized with the bi-layer organic (Figure 4d), a common feature of the proposed bi-layer organic heterostructures is the re-absorption of the emitted radiation. Therefore, the loss of incident radiation through PL in the L13, L78, and ZnPc layers by radiative processes could be limited, adding the supplementary layer of TPyP. Thus, the radiation emitted by the first layer (ZnPc, L13, L78) could be partially reabsorbed by the second layer (TPyP) on a deep energetic level and could be involved in the generation of charge carriers.

**Figure 9:**
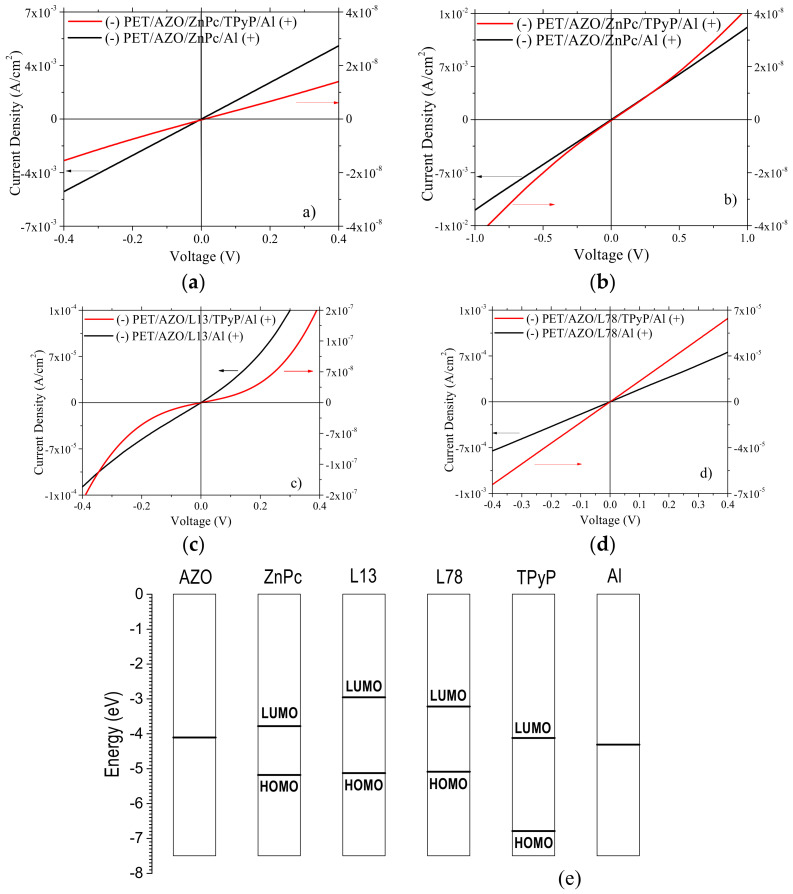
Typical J-V characteristics at room temperature in dark for organic heterostructures with single- and bi-layer organics on PET substrate and AZO and Al electrodes (**a**–**d**). The polarization of the heterostructures is indicated in the figure. The substrates are indicated in Table 1. Energy levels diagram for each component of the investigated organic heterostructures (**e**).

Most of the heterostructures realized on the flexible substrate with single- or bi-layer organic and AZO, and Al electrodes showed a good injection of the charge carriers at low voltages < 0.4 V: linear J-V characteristics (Figure 9a,d) or non-linear J-V characteristic (Figure 9c), where J = current density. The non-linear behavior is likely determined by the intrinsic surface and interface effects [81]. All the heterostructures with a single organic layer are characterized by a good value of the current (>10^−5^ A). The highest current density at a low applied voltage of 0.3 V has been obtained in the heterostructures with a single organic layer of ZnPc, J ~3.93 mA/cm^2^ (Figure 9a), and L78, J ~0.57 mA/cm^2^ (Figure 9d).

The effect of the PET substrate could be important because the substrate’s surface topography affects the morphology of the subsequently deposited layers. Analyzing the heterostructures prepared with a single organic film, we remarked that the energy balance dominates over the morphology. Thus, the layer of L78 presents a large grain morphology with a smaller number of grain boundaries for charge carriers scattering or recombination. However, the highest current density was obtained with the ZnPc layer showing smaller grain morphology and an increased number of grain boundaries determining the scattering of the charge carriers. The following values have been used for the position of the energetic levels of the components of the heterostructures (Figure 9e): the work function of AZO (WF_AZO_ = 4.1 eV [82]) and Al (WF_Al_ = 4.3 eV [83]) and the position of HOMO and LUMO levels in ZnPc (E_HOMO;ZnPc_ = 5.17 eV [39] and E_LUMO;ZnPc_ = 3.78 eV [39]), L13 (E_HOMO;L13_ = 5.14 eV and E_LUMO;L13_ = 2.95 eV evaluated by cyclic voltammetry [38], and L78 (E_HOMO;L78_ = 5.1 eV and E_LUMO;L78_ = 3.21 eV evaluated by cyclic voltammetry [38]).

When AZO is negatively polarized, and Al is positively polarized, the charge carriers must surpass lower energetic barriers in the heterostructure realized with a single ZnPc layer (ΔE_LUMO,ZnPc-WF,AZO_ = 0.32 eV) compared to the heterostructures realized with arylenevinylene oligomer L13 or L78 (ΔE_LUMO,L13-WF,AZO_ = 1.15 eV and ΔE_LUMO,L78_-_WF, AZO_ = 0.89 eV). Therefore, we expected a better injection and transport of the charge carriers in the heterostructure with the ZnPc film. Experimentally, the highest current density was obtained in the heterostructure PET/AZO/ZnPc/Al, which is characterized by a lower energetic barrier at the interface AZO/organic semiconductor (Figure 9d) than the heterostructures prepared with L78 and L13 single films. This behavior confirms the theoretical prediction.

The most important effects induced by the supplementary layer of TPyP on the electrical properties of the heterostructures realized on PET covered by AZO is the change in the shape of the J-V characteristic and decrease in the value of the current density. Thus, the J-V characteristic change from slightly non-linear asymmetric to non-linear almost symmetrical for PET/AZO/L13 (Figure 9c) at applied voltage < 0.4 V. A change of J-V characteristic from linear to slightly symmetrical non-linear has been revealed for the heterostructure PET/AZO/ZnPc (Figure 9b) only for higher applied voltages (> 0.4 V). Independently of the donor layer (ZnPc, L13, L78), by adding a TPyP film, the current density decreased with orders of magnitude for applied voltage < 0.4 V (Figure 9a,c,d). The lowest decrease was obtained by adding a layer of TPyP at the heterostructure realized with the L78 layer (Figure 9d).

The height of the energetic barrier at the interface ZnPc/TPyP, L13/TPyP and L78/TPyP (E_LUMO;TPyP_ = 4.1 eV [16,84]) cannot justify the above mentioned behavior. To explain the behavior of these organic heterostructures, we must consider factors related to the electronic properties of the triphenylamine-based oligomer L78 [14], favoring the electrons easy transfer from the donor (L78) to the acceptor (TPyP). Additionally, the current in the heterostructures was determined by the contribution to the resistivity of the surface through surface roughness and grain boundaries [85]. Thus, the morphological and topographical particularities of the organic layer are also important. The TPyP film deposited on top of the PET/AZO/L78 (Figure 6f, Table 3) shows larger grains/clusters and higher roughness than the TPyP film deposited on top of the PET/AZO/ZnPc (Figure 6d, Table 3) and PET/AZO/L13 (Figure 6e, Table 3). The higher roughness could be associated with a higher electrical resistance of the contact between the TPyP deposited on the L78 and Al electrodes. However, larger grains revealed by AFM images mean a reduced number of grain boundaries, which determines a weaker scattering of the charge carriers in the L78/TPyP bi-layer heterostructure, with a positive effect on the current. On the other hand, the increased contact area determined by the lower surface roughness of the TPyP film deposited on ZnPc or L13 sustains a lower resistance of the electrical contact between TPyP and Al electrode, which might improve the injection and collection of the charge carriers. On the contrary, the experimental data have revealed lower currents, with orders of magnitude, for these heterostructures. This means that the effect of the grain boundaries on the charge carriers’ loss inside the organic layers became dominant over the surface roughness. This is the result of the charge carriers’ increased scattering on the higher number of grain boundaries characterizing the small grain morphology, which strongly counteracted the positive effect of the low roughness. This mechanism is dominant in the case of smoother surfaces (TPyP on ZnPc and TPyP on L13), determining the decrease in the current. The experimental data obtained with our heterostructures confirmed that the large grain morphology was dominant over the reduction of the electrical contact resistance for smoother surfaces.

The electrical properties of the thin films can be strongly affected by the cross-correlation of the roughness effects [86]. It has already been demonstrated theoretically that the transport properties of the semiconducting films depend on the electron scattering on the surface roughness and are determined by the roughness exponent, α [87]. The conductivity increases with increasing α because it decreases the scattering by roughness (smooth surface are characterized by α close to 1) [87]. Thus, a high α, between 0.8 and 1, characterizes rounded surface grains (α = 1 correspond to flat grain [87]). In our case, the current increases from the heterostructure with L78 to heterostructure with TPyP on L78 (Figure 9d) because α increases from 0.8 to 0.85 (Figure 7c,f). Sharp conic and pyramidal grain morphology was shown by the layers of ZnPc and L13 deposited on PET/AZO, for which the α was around 0.7 (Figure 7a,b). Thus, we expect a lower conduction for the sample with a lower roughness exponent (meaning rougher surfaces) [87]. These grains became sharper α < 0.7, when TPyP was deposited on ZnPc (Figure 7d), and the conductivity decreased by an order of magnitude (Figure 9a,b). The layer of TPyP deposited on L13 is also characterized by α > 0.8 (Figure 7e) associated with a relatively smooth surface and good conductivity (Figure 9c). Therefore, the layer of TPyP deposited on L13 showed slightly better conduction (Figure 9c) than the layer of TPyP deposited on ZnPc (Figure 9a). The conduction was favored in the layer characterized by round (L78), close to flat (TPyP on L78), grain morphology.

Hence, the electrical properties of the organic heterostructures are the result of the balance between different counteracting phenomena. Thus, the scattering of the charge carriers on the grain boundaries affects the transport inside the layer. The contact area between the organic and electrode determined by the surface topography and surface roughness, affects the contact resistance and the charge carrier injection/collection. Consequently, the surface roughness (indicated by the roughness exponent α and surface amplitude parameter RMS) and grain shape explain the electrical behavior of the heterostructures with arylenevinylene oligomers and non-metallic porphyrin-stacked layers. The highest density of current for an applied voltage of 0.3 V has been obtained with a bi-layer heterostructure L78/TPyP deposited on PET/AZO, J = 0.97 mA/cm^2^ compared to ZnPc/TPyP, J = 9.14 × 10^−6^ mA/cm^2^ and L13/TPyP, J = 11.68 × 10^−5^ mA/cm^2^. Thus, the oligomer based on triphenylamine, L78, is a promising candidate for applications in flexible electronics.

## 4. Conclusions

We propose a new type of bi-layer organic, flexible heterostructure based on arylenevinylene compounds, carbazole (L13), and triphenylamine (L78), instead of zinc phthalocyanine (ZnPc), as a donor and a non-metallic porphyrin (TPyP) as an acceptor. The heterostructures have been realized by vacuum evaporation on Al-doped ZnO (AZO) films showing high transparencies ~90%, over the visible range and resistivity between 6.9 × 10^−4^ Ω·cm and 23 × 10^−4^ Ω·cm, deposited by PLD on PET. The AZO films deposited on PET showed a morphology characterized by grains or clusters of grains and defects (nonuniformities), which have been evidenced by SEM and confirmed by AFM. XRD measurements have revealed a slight preferential orientation of some grains in the AZO film deposited on the PET in a direction perpendicular to the substrate, while most of the grains were randomly oriented.

The bi-layer heterostructures based on the arylenevinylene oligomer as the donor and TPyP as the acceptor showed many absorption peaks over the visible domain, most of them associated with the presence of the well-defined absorption bands of TPyP. The supplementary layer of TPyP can reduce the loss of radiation by photoluminescence emission through the re-absorption of the radiation emitted by the first layer (ZnPc, L13, L78), thus favoring the charge carrier generation.

The particularities of the J-V characteristics have been discussed as the result of the balance between factors related to energetic barriers, layer morphology, and surface topography of the component layers. Thus, the heterostructures realized on PET with a single-layer organic between the AZO and Al electrodes have shown an injection contact behavior with the J-V characteristic either linear (ZnPc, L78) or non-linear (L13) at low voltages (<0.4 V). A supplementary layer of TPyP has determined a decrease in the current density with orders of magnitudes compared to the heterostructure with a single layer. This behavior is explained by the roughness exponent, α, of the surfaces evaluated using the height-height correlation function and the associated grain shapes. The most efficient injection of the charge carriers and higher density of current, J~1 mA/cm^2^, has been obtained in the bi-layer heterostructure PET/AZO/L78/TPyP/Al. Therefore, the arylenevinylene oligomer based on triphenylamine, L78, is a promising donor to be used together with the TPyP acceptor layer in flexible electronic applications.

## Figures and Tables

**Table 1:** Investigated heterostructures and the main properties of the AZO layers.

Heterostructure	Substrate/AZO	AZO Thickness * (nm)	AZO Resistivity * (×10^−4^ Ω·cm)
PET/AZO/ZnPc/Al	P1	340 ± 30	7.3 ± 0.6
PET/AZO/L13/Al	P3	390 ± 70	23 ± 4
PET/AZO/L78/Al	P5	330 ± 80	8.2 ± 1.9
PET/AZO/ZnPc/TPyP/Al	P2	300 ± 60	6.9 ± 1.5
PET/AZO/L13/TPyP/Al	P4	450 ± 50	11 ± 1.2
PET/AZO/L78/TPyP/Al	P6	490 ± 150	8.6 ± 2.5

* Error is the confidence interval (standard uncertainty σ).

**Table 2:** Elemental composition of Al-doped ZnO films evaluated by EDX measurement.

Sample	Target Composition	Element	Concentration * [at. %]
PET/AZO	2 wt % Al in ZnO	Aluminum	1.8 ± 0.1
		Zinc	23.0 ± 1.6
		Oxygen	55.4 ± 4.3

* Error is the confidence interval (standard uncertainty σ).

**Table 3:** Surface amplitude parameters of the single- and bi-layer organic deposited on PET/AZO. For PET and PET/AZO, the RMS and RA are the average values for six samples.

Sample	RMS (nm)	RA (nm)
PET	11.2	7.3
PET/AZO	13.5	9.6
PET/AZO/ZnPc	12.3	8.3
PET/AZO/L13	22.0	13.6
PET/AZO/L78	43.0	31.0
PET/AZO/ZnPc/TPyP	8.0	6.2
PET/AZO/L13/TPyP	13.0	10.2
PET/AZO/L78/TPyP	61.0	46.0

## Data Availability

The data presented in this study are available on request from the corresponding author.
